# Echocardiography‐defined pulmonary hypertension is an adverse prognostic factor for newly diagnosed multiple myeloma patients

**DOI:** 10.1002/cam4.4770

**Published:** 2022-04-24

**Authors:** Yuan Jian, Huixing Zhou, Yidan Wang, Zhiyao Zhang, Guangzhong Yang, Chuanying Geng, Ying Tian, Wen Gao, Wenming Chen

**Affiliations:** ^1^ Department of Hematology, Myeloma Research Center of Beijing Beijing Chaoyang Hospital, Capital Medical University Beijing China; ^2^ Department of Cardiology Beijing Chaoyang Hospital, Capital Medical University Beijing China

**Keywords:** echocardiography, multiple myeloma, prognosis, pulmonary hypertension, survival

## Abstract

**Background:**

Pulmonary hypertension (PH) is a common but rarely recognized comorbidity of multiple myeloma (MM) patients, while its prognostic significance for MM has been rarely reported.

**Methods:**

We retrospectively analyzed the clinical characteristics and prognostic value of baseline echocardiography‐defined PH in 426 newly diagnosed MM (NDMM) patients.

**Results:**

Echocardiograph‐defined PH was found in 12.7% (54/426) of NDMM patients, associated with older age, anemia, and renal insufficiency, as well as severe diastolic dysfunction and higher BNP and NT‐pro‐BNP levels. Patients with PH presented with a higher prevalence of atrial fibrillation, while with a similar incidence of thrombosis compared with those without PH. Based on similar treatment regimens and autologous stem cell transplantation (ASCT) rates, patients without PH have deeper and better responses than those with PH (*p* = 0.002). With the remission of MM, 81.5% of PH was reversible, accompanied by improvement of right ventricular dysfunction and normalization of BNP/NT‐pro‐BNP levels, while could reoccur at MM relapse. Survival analysis revealed that PH was an adverse prognostic factor, associated with reduced progression‐free survival (PFS) (21 vs. 50 months, *p* < 0.001) and overall survival (OS) (45 vs. 90 months, *p* = 0.014). Multivariate analysis further verified that baseline PH was an independent predictor for shorter PFS and OS.

**Conclusion:**

In conclusion, echocardiography‐defined PH is an adverse prognostic indicator for MM patients and should be routinely evaluated in MM patients at diagnosis to make a precise prognosis.

## Graphical abstract

Pulmonary hypertension (PH) is an adverse prognostic factor of progression‐free survival (PFS) and overall survival (OS) for newly diagnosed multiple myeloma patients.

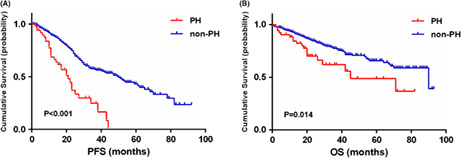

## 1 INTRODUCTION

Multiple myeloma (MM) is one of the most common hematological malignancies characterized by the clonal proliferation of plasma cells and usually presented with anemia, hypercalcemia, bone lesions, and renal insufficiency.^1^ MM is more frequently in the elderly, with a median diagnosis age of 70 years.^2^ The high incidence of cardiovascular comorbidities in MM has been reported,^3^ which might be attributed to age‐related cardiac risk factors, disease‐related risk factors such as amyloidosis, hyperviscosity, and anemia, as well as treatment‐related factors.^4^ Common recognized cardiovascular comorbidities in MM patients include cardiac arrhythmia, ischemic heart disease, and chronic heart failure.^3^ The existence of these comorbidities enhances treatment‐related toxicities and is usually associated with decreased survival.

Pulmonary hypertension (PH) is a composite of various pathological conditions presented with elevated pulmonary arterial pressure (PAP), predominantly in cardiovascular diseases.^5^ PH is not a commonly recognized comorbidity in MM. Although its prevalence has rarely been reported, the coexistence of PH and MM has been found in recent years.^4^, ^6^, ^9^ According to two retrospective studies, complicating PH in MM could result in elevated cardiovascular toxicity and relatively high mortality.^8^, ^9^ Possible mechanisms of PH in MM include amyloidosis depositions and treatments of MM such as thalidomide and carfilzomib, which could explain the etiology of PH in some specific cases.^6^, ^7^, ^10^ However, for the majority of newly diagnosed MM (NDMM) patients without amyloidosis, the mechanism of baseline PH is still unclarified, while the reports of prevalence and prognosis value of PH are quite limited.^8^ Therefore, the present study was designed to evaluate the clinical characteristic and prognostic value of echocardiography‐defined PH in newly diagnosed MM (NDMM) patients and to explore the potential mechanisms.

## 2 MATERIALS AND METHODS

### 2.1 Patients

A total of 426 symptomatic MM patients from Beijing Chaoyang Hospital (Beijing, China) who were first diagnosed between January 2014 and December 2020 were enrolled in this study. Patients were required to have available baseline echocardiography results at MM diagnosis before receiving antimyeloma treatment. The diagnosis of MM was based on the International Myeloma Working Group (IMWG) criteria.^11^ Patients with cardiac light chain amyloidosis were excluded from the analysis. Specific screening methods were as follows: bone marrow (BM) biopsy and Congo red staining were routinely performed in all patients. For those with negative BM biopsy while presenting with suspected amyloidosis characteristics, biopsy and Congo red staining of other tissues such as tongue, abdominal fat, and kidney would be repeated. For those with a thickened interventricular septum, cardiac magnetic resonance imaging (MRI) would be performed. Patients presenting with positive Congo red staining in any tissue biopsy or cardiac amyloidosis MRI profiles would be excluded from our study.

Induction regimens contained bortezomib‐based regimens (*n* = 285), lenalidomide‐based regimens (with or without bortezomib, *n* = 110), ixazomib‐based regimens (*n* = 1), daratumumab‐based regimens (*n* = 4), and traditional chemotherapy regimens (*n* = 26). Autologous stem cell transplantation (ASCT) was administrated in 145 patients. Response to therapy was evaluated according to IMWG criteria.^12^ The cutoff date of follow‐up was 31 October 2021. The median follow‐up time was 40 (10–93) months. This study was approved by the Ethics Committee of Beijing Chaoyang Hospital. Written informed consents were obtained from all patients.

### 2.2 Echocardiography examination

Echocardiography examination was administrated using three different echocardiography equipment systems (Vivid 7 and Vivid E9, GE Vingmed Ultrasound, Horten, Norway; Philips IE33, Philips Healthcare). A series of parameters were measured including left ventricular end‐diastolic and end‐systolic diameter (LVEDD and LVESD, respectively), interventricular septum diameter (IVSD), left ventricular posterior wall thickness diameter (LVPWD), left atrial anteroposterior, left and right, and superoinferior diameter (LAAPD, LALRD, and LASID, respectively), left ventricular ejection fraction (LVEF), right ventricle diameter (RVD), pulmonary artery diameter (PAD), also the early and late diastolic velocity of mitral inflow (E wave and A wave).^13^ Diastolic dysfunction was defined as Grades 1, 2, and 3 according to the guidelines of the American Society of Echocardiography.^14^ The modified Bernoulli formula was employed to estimate systolic pulmonary arterial pressure (sPAP).^14^ Based on the literature, PH was defined as sPAP >40 mmHg,^15^ whereas sPAPs below 35 mmHg were not reported as a specific value.

### 2.3 Statistical analysis

Categorical clinical characteristics were described as percentages and were compared using the *χ* ^2^ test or two‐sided Fisher exact test between the groups. Continuous clinical characteristics and echocardiographic parameters were described as median and range or median ± standard deviation and were compared using Wilcoxon rank‐sum tests between the groups. PAPs between different disease statuses were compared by Wilcoxon rank‐sum tests of paired samples.

Progression‐free survival (PFS) was defined as the duration from the initiation of treatment to the date of the first progression or death from any cause. Overall survival (OS) was defined as the duration from the initiation of treatment to death from any cause.^16^ Survival curves were plotted using the Kaplan–Meier method and the differences between curves were assessed by the log‐rank test. The prognostic value of the factors was evaluated by the Cox proportional hazard regression analysis. All statistical analyses were performed using SPSS version 24.0 (SPSS, Inc). Statistical significance was considered if the *p* value was <0.05.

## 3 RESULTS

### 3.1 Clinical characteristics of PH

Echocardiograph‐defined PH was found in 12.7% (54/426) of NDMM patients. Median PAP was 46.6 mmHg (40.3–69.0 mmHg) among patients with PH. The clinical baseline characteristics and therapy types between PH and non‐PH patients were evaluated in all the 426 patients (Table 1). Results revealed that age, hemoglobin, serum creatinine, and β2‐microglobulin were associated with PH. Patients with PH were presented with older age (64 vs. 61 years old, *p* = 0.006), lower hemoglobin concentration (85 vs. 95 g/L, *p* = 0.002), higher serum creatinine level (118.2 vs. 78.7 μmol/L, *p* < 0.001), and β2‐microglobulin concentration (10.12 vs. 5.06 mg/L, *p* < 0.001) than those without PH. Although serum D‐dimer values were higher in patients with PH (1.11 vs. 0.67 mg/L, *p* = 0.049), the difference did not reach statistical significance compared with age‐adjusted D‐dimer cutoff levels, which is defined as age ×10 (μg/L) in patients 50 years or older and a fixed value of 500 μg/L in patients under 50 years old.^17^ Other clinical characteristics including gender, disease stages (DS, ISS, and R‐ISS), M component, high‐risk cytogenetics, corrected calcium, lactate dehydrogenase (LDH), and albumin did not show statistical differences between the two groups (*p* > 0.05) (Table 1). Two patients in PH group and 18 patients in the non‐PH group had evidence of deep venous thrombosis (DVT) at MM diagnosis, which did not show statistical differences (*p* = 0.762). Among these thrombosis patients, two had been diagnosed with pulmonary embolism (PE), who were both in the non‐PH group (*p* = 1.000). The proportion of patients receiving novel‐agent induction regimens and autologous stem cell transplantation (ASCT) was comparable between the PH and non‐PH patients, which did not show statistical differences.

**TABLE 1:** Correlation between pulmonary hypertension (PH) and clinical characteristics

*n* = 426	Patients with PH (*n* = 54)	Patients without PH (*n* = 372)	*p* value
Gender			0.756
Male	30/54 (55.6)	215/372 (57.8)	
Female	24/54 (44.4)	157/372 (42.2)	
Age (years)	64 (45–84)	61 (28–85)	0.006^**^
DS stage			0.255
I	0/54 (0.0)	18/372 (4.8)	
II	8/54 (14.8)	54/372 (14.5)	
III	46/54 (85.2)	300/372 (80.6)	
ISS stage			0.051
I	4/54 (7.4)	71/372 (19.1)	
II	14/54 (25.9)	110/372 (29.6)	
III	36/54 (66.7)	191/372 (51.3)	
R‐ISS stage			0.061
I	1/50 (2.0)	48/350 (13.7)	
II	36/50 (72.0)	220/350 (62.9)	
III	13/50 (26.0)	82/350 (23.4)	
M component			0.230
IgG	21/54 (38.9)	172/372 (46.2)	
IgA	12/54 (22.2)	76/372 (20.4)	
IgD	0/54 (0.0)	20/372 (5.4)	
Light chain	18/54 (33.3)	92/372 (24.7)	
Nonsecretory	3/54 (5.6)	12/372 (3.2)	
High‐risk cytogenetics^†^	15/46 (32.6)	102/337 (30.3)	0.746
Hemoglobin (g/L)	85 (29–152)	95 (40–163)	0.002^**^
Corrected calcium (mmol/L)	2.38 (1.96–4.14)	2.34 (1.67–4.08)	0.091
Lactate dehydrogenase (U/L)	182 (80–853)	163 (20–1580)	0.065
Serum creatinine (μmol/L)	118.2 (41.1–865.2)	78.7 (30.0–1436.6)	<0.001^***^
Albumin (g/L)	34.5 (19.6–50.3)	35.2 (12.5–51.2)	0.834
β2‐microglobulin (mg/L)	10.12 (0.55–58.20)	5.06 (1.39–67.6)	<0.001^***^
D‐dimer (mg/L)	1.11 (0.10–20.84)	0.67 (0.10–29.77)	0.049^*^
Age‐adjusted D‐dimer cutoff^§^	34/51 (66.7%)	189/344 (54.9)	0.131
Thrombosis	2/54 (3.7%)	18/372 (4.8%)	0.762
Novel‐agent induction regimens	49/54 (90.7)	351/372 (94.4)	0.300
ASCT^‡^	15/54 (27.8)	135/372 (36.3)	0.222

*Note*: Data are presented as n (%) or median (range).

^†^ High‐risk cytogenetics: del(17p), t(4;14), and/or t(14;16).

^‡^ ASCT: autologous stem cell transplantation.

^§^ Age‐adjusted D‐dimer cutoff, defined as age ×10 (μg/L) in patients 50 years or older and a fixed value of 500 μg/L in patients under 50 years old.

^*^ *p*< 0.05

^**^ *p*< 0.01

^***^ *p*< 0.001.

Since various cardiovascular and lung diseases are also known to be related to PH, baseline prevalence of these comorbidities and existing risk factors of cardiovascular diseases were also investigated between the two groups to further understand the pathogenesis and characteristics of PH (Table 2). According to the results, the incidence of atrial fibrillation (AF) was significantly higher in the PH group than in the non‐PH group (9.3% vs. 0.8%, *p* = 0.001). No statistical differences were shown between the prevalence of other diseases including coronary heart disease, chronic heart failure, valvular heart disease, and chronic obstructive pulmonary disease (COPD) in the two groups. Moreover, the incidences of some well‐known risk factors for these cardiovascular diseases, including hypertension, hyperlipidemia, diabetes, and smoking, were also comparable between groups (Table 2).

**TABLE 2:** Correlation between pulmonary hypertension (PH) and prevalence of baseline cardiovascular and lung conditions

*n* = 426	Patients with PH (*n* = 54)	Patients without PH (*n* = 372)	*p* value
Baseline cardiovascular and lung diseases			
Coronary heart disease	8/54 (14.8)	29/372 (7.8)	0.115
Chronic heart failure	1/54 (1.9)	2/372 (0.5)	0.335
Atrial fibrillation	5/54 (9.3)	3/372 (0.8)	0.001^**^
Valvular heart disease	0/54 (0.0)	1/372 (0.3)	1.000
COPD^†^	2/54 (3.7)	6/372 (1.6)	0.269
Total	13/54 (24.1)	36/372 (9.7)	0.002^**^
Risk factors for cardiovascular diseases			
Hypertension	25/54 (46.3)	162/372 (43.5)	0.704
Hyperlipidemia	2/54 (3.7)	37/372 (9.9)	0.204
Diabetes	8/54 (14.8)	50/372 (13.4)	0.783
Smoking	16/54 (29.6)	100/372 (26.9)	0.672
Total	34/54 (63.0)	239/372 (64.2)	0.854

*Note*: Data are presented as n (%) or median (range).

^†^ COPD, chronic obstructive pulmonary disease.

^**^ *p* < 0.01.

### 3.2 Echocardiographic characteristics and cardiac parameters of PH

Echocardiographic characteristics were compared between PH and non‐PH patients (Table 3). Results showed that patients with PH had higher left atrial diameters (LAD), including anteroposterior, left and right, and superoinferior diameters (*p* < 0.001), as well as severe diastolic dysfunction (*p* < 0.001). Other parameters, including LVEDD, LVESD, LVPWD, RVD, and PAD, were all statistically higher in patients with PH than those without PH while were not beyond clinical normal levels.

**TABLE 3:** Echocardiographic characteristics and cardiac parameters between PH and non‐PH patients

*n* = 426	Patients with PH (*n* = 54)	Patients without PH (*n* = 372)	*p*value
LVEDD (mm)	50 (38–59)	47 (36–63)	0.001^**^
LVESD (mm)	30 (22–39)	28 (16–50)	0.030^*^
IVSD (mm)	10.0 (7.0–14.0)	10.0 (6.0–14.5)	0.155
LVPWD (mm)	9.9 (7.0–12.5)	9.0 (6.4–15.0)	0.042^*^
LAD			
LAAPD (mm)	39 (30–50)	35 (23–60)	<0.001^***^
LALRD (mm)	42 (28–57)	37 (25–61)	<0.001^***^
LASID (mm)	53 (41–68)	48 (28–70)	<0.001^***^
LVEF (%)	67 (45–82)	69 (49–87)	0.069
Mitral valve peak velocity			
E wave (cm/s)	108 (52–170)	83 (39–216)	<0.001^***^
A wave (cm/s)	90 (44–152)	92 (38–181)	0.592
E/A ratio	1.17 (0.67–3.21)	0.85 (0.47–2.95)	0.061
RVD (mm)	35 (26–45)	32 (21–43)	<0.001^***^
PAD (mm)	25 (20–32)	24 (15–37)	<0.001^***^
Diastolic dysfunction			<0.001^***^
Grade 1	0/54 (0.0)	269/372 (72.3)	
Grade 2	34/54 (63.0)	5/372 (1.3)	
Grade 3	6/54 (11.1)	5/372 (1.3)	
BNP (pg/ml)	159 (6–>5000)	50 (2–2497)	<0.001^***^
NT‐pro‐BNP (pg/ml)	906.1 (91.9–24888.0)	119.3 (12.3–18,723)	<0.001^***^

*Note*: Data are presented as n (%) or median (range).

Abbreviations: A wave, late diastolic velocity of mitral inflow; E wave, early diastolic velocity of mitral inflow; IVSD, interventricular septum diameter; LAAPD, left atrial anteroposterior diameter; LAD, left atrial diameter; LALRD, left atrial left and right diameter; LASID, left atrial superoinferior diameter; LVEDD, left ventricular end‐diastolic diameter; LVEF, left ventricular ejection fraction; LVESD, left ventricular end‐systolic diameter; LVPWD, left ventricular posterior wall thickness diameter; NT‐pro‐BNP, N‐terminal pro‐brain natriuretic peptide; PAD, pulmonary artery diameter. BNP, brain natriuretic peptide; PH, pulmonary hypertension; RVD, right ventricle diameter.

^*^ *p* < 0.05

^**^ *p* < 0.01

^***^ *p* < 0.001.

Among all 426 patients, 409 of them had available baseline brain natriuretic peptide (BNP) or N‐terminal pro‐BNP (NT‐pro‐BNP) levels. Due to the transformation of laboratory panels, NT‐pro‐BNP levels were detected in 169 patients before September 30, 2017, while BNP levels were detected in 240 patients after October 1, 2017. These two cardiac biomarkers were compared separately between PH and non‐PH patients (Table 3). Results showed that both BNP and NT‐pro‐BNP levels were significantly higher in patients with PH (*p* < 0.001).

### 3.3 PHand response rate

Among all 426 patients, 390 patients had the available evaluable best response to induction therapy or ASCT. The correlation between PH and response rate in these patients was investigated (Table 4). According to the results, patients without PH have deeper and better responses than those with PH (*p* = 0.002). The overall response rate in patients with PH was 85.4%, comparing with those without PH being 94.7%. Moreover, the complete response (CR) and stringent complete response (sCR) rate in patients with PH (33.3%) was lower than in those without PH (52.3%). Taking into consideration that the two groups had comparable treatment strategies including ASCT rates (Table 1), these results indicated that patients with PH were less likely to get deeper responses than those without PH.

**TABLE 4:** Response rate according to PH

Response	Patients with PH (*n* = 48)	Patients without PH (*n* = 342)
sCR	5/48 (10.4)	105/342 (30.7)
CR	11/48 (22.9)	74/342 (21.6)
VGPR	14/48 (29.2)	77/342 (22.5)
PR	11/48 (22.9)	68/342 (19.9)
SD/PD	7/48 (14.6)	18/342 (5.3)

*Note*: Data are presented as *n* (%).

Abbreviations: CR, complete response; PD, progressive disease; PH, pulmonary hypertension; PR, partial response; sCR, stringent complete response; SD, stable disease; VGPR, very good partial response.

### 3.4 PHand disease status

**FIGURE 1:**
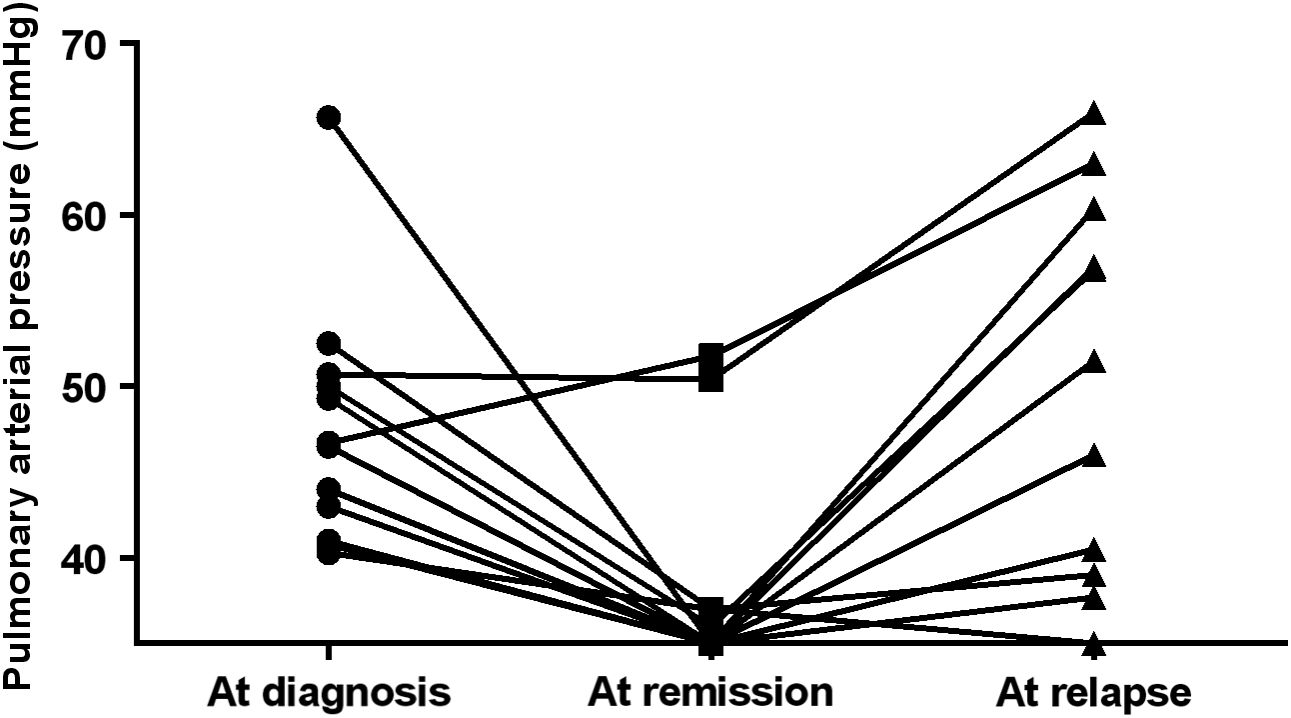
Pulmonary arterial pressures (PAPs) on different disease statuses

Among the 54 patients with PH at baseline, 36 of them had reexamined echocardiography at different disease statuses. By comparing the time of echocardiography examinations and disease status, a total of 27 patients had reached at least partial response (PR) while they had available echocardiography results at remission, whereas 14 of them had available entire series of echocardiography results at MM diagnosis, remission (≥PR), and relapse. PAPs at different disease statuses were compared among these patients. Results revealed that with the remission of MM, PAPs of 81.5% (22/27) patients had returned to the normal range (<40 mmHg). Meanwhile, improvement of right ventricular dysfunction was seen in 68.8% (11/16) patients with evaluable diastolic dysfunction grade, while normalization of BNP/NT‐pro‐BNP was seen in 52.2% (12/23) patients with elevated baseline BNP/NT‐pro‐BNP levels at MM remission. Among the 14 patients with an entire series of echocardiography results in different disease statuses, results showed that PAPs were significantly lower at remission (median < 35 mmHg) than at diagnosis (median 45.3 mmHg) while were elevated again at MM relapse (median 43.3 mmHg, *p* = 0.002) (Figure 1).

### 3.5 Survival analysis

**FIGURE 2:**
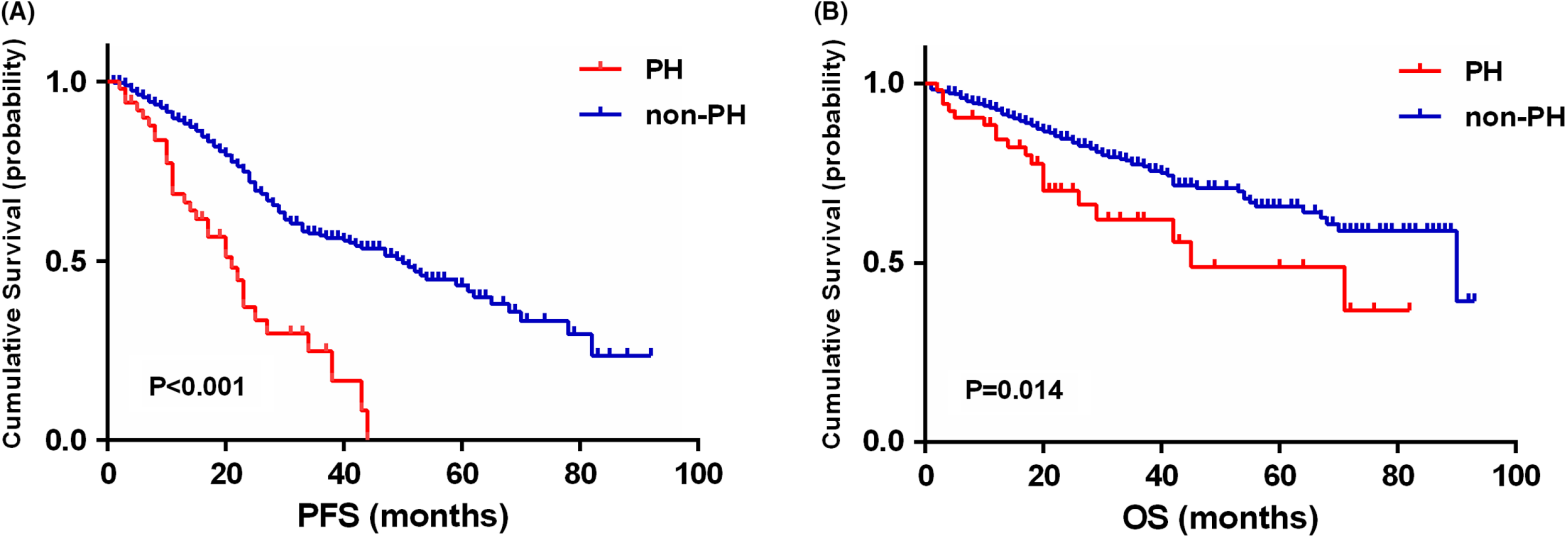
Effects of PH on progression‐free (PFS) (A) and overall survival (OS) (B)

The impact of PH on PFS and OS in MM patients was assessed by survival analysis. According to the results, patients with PH had shorter PFS and OS than those without PH. The median PFS in patients with PH was 21.0 months (95% confidence interval, CI: 14.7–27.3), whereas in those without PH, it was 50.0 (95% CI: 40.8–59.2) months (*p* < 0.001) (Figure 2A). A similar tendency was also shown in OS. The median OS in patients with PH was 45.0 (95% CI: 14.0–76.0) months, whereas in non‐PH patients, it was 90.0 (95% CI: 57.4–122.6) months (Figure 2B) (*p* = 0.014).

### 3.6 Multivariate analysis

The influence of PH and other clinical parameters on PFS and OS were tested in univariate and multivariate analyses. In univariate analysis, ISS stage III, corrected calcium ≥2.6 mmol/L, serum creatinine ≥130 μmol/L, LDH ≥250 U/L, and PH was associated with shorter PFS, whereas age ≥ 65 years, ISS stage III, corrected calcium ≥2.6 mmol/L, serum creatinine ≥130 μmol/L, hemoglobin <90 g/L, LDH ≥250 U/L, high‐risk cytogenetics, non‐ASCT, and PH were also associated with shorter OS (Table 5). Parameters with low P values (<0.1) in the univariate analysis were subsequently examined in multivariate analysis. According to the results, PH retained its negative impact on both PFS (harzard ratio, HR: 2.901, 95% CI: 1.812–4.643, *p* < 0.001) and OS (HR: 1.903, 95% CI: 1.050–3.451, *p* = 0.034). Other significant factors in multivariate analysis included ISS stage III (HR: 1.518, 95% CI: 1.019–2.260, *p* = 0.040) and LDH ≥250 U/L (HR: 1.891, 95% CI: 1.189–3.007, *p* = 0.007), which were independent indicators for shorter PFS, whereas corrected calcium ≥2.6 mmol/L (HR: 2.019, 95% CI: 1.195–3.412, *p* = 0.009) and LDH ≥250 U/L (HR: 3.065, 95% CI: 1.868–5.030, *p* < 0.001) were independent indicators for shorter OS. In addition, ASCT was independent indicator for both longer PFS (HR: 0.622, 95% CI: 0.427–0.907, *p* = 0.014) and OS (HR: 0.415, 95% CI: 0.231–0.744, *p* = 0.003) (Table 6).

**TABLE 5:** Univariate analysis of variables associated with patient outcomes

Parameter	PFS			OS		
	HR	95% CI	*p*value	HR	95% CI	*p*value
Age (years) ≥65 vs. <65	1.061	0.772–1.458	0.714	1.649	1.127–2.411	0.010^*^
ISS stage III vs. I–II	1.915	1.400–2.620	<0.001^***^	2.320	1.541–3.492	<0.001^***^
Corrected calcium (mmol/L) ≥ 2.6 vs. < 2.6	1.446	1.007–2.078	0.046^*^	2.004	1.320–3.043	0.001^**^
Serum creatinine (μmol/L) ≥ 130 vs. < 130	1.513	1.090–2.100	0.013^*^	1.653	1.116–2.449	0.012^*^
Hemoglobin (g/L) <90 vs. ≥90	1.222	0.898–1.663	0.202	1.761	1.198–2.590	0.004^**^
Lactate dehydrogenase (U/L) ≥250 vs. <250	1.855	1.229–2.800	0.003^**^	2.803	1.832–4.289	<0.001^***^
High‐risk cytogenetics^†^ Positive vs. negative	1.389	0.997–1.936	0.052	1.526	1.007–2.313	0.046^*^
ASCT Yes vs. no	0.734	0.535–1.007	0.056	0.595	0.394–0.898	0.014^*^
PH Yes vs. no	2.986	2.014–4.425	<0.001^***^	1.839	1.119–3.020	0.016^*^

Abbreviations: ASCT, autologous stem cell transplantation; CI, confidence interval; HR, harzard ratio; PH, pulmonary hypertension.

^†^ High‐risk cytogenetics: del(17p), t(4;14), and/or t(14;16).

^*^ *p* < 0.05

^**^ *p* < 0.01

^***^ *p* < 0.001.

**TABLE 6:** Multivariate analysis of variables associated with patient outcomes

Parameter	PFS			OS		
	HR	95% CI	*p*value	HR	95% CI	*p*value
Age (years) ≥65 vs. <65	‐	‐	‐	1.164	0.706–1.920	0.552
ISS stage III vs. I‐II	1.518	1.019–2.260	0.040^*^	1.575	0.904–2.744	0.109
Corrected calcium (mmol/L) ≥ 2.6 vs. < 2.6	1.456	0.938–2.259	0.094	2.019	1.195–3.412	0.009^**^
Serum creatinine (μmol/L) ≥130 vs. <130	0.945	0.629–1.419	0.785	0.912	0.551–1.509	0.719
Hemoglobin (g/L) <90 vs. ≥90	‐	‐	‐	1.529	0.956–2.446	0.076
Lactate dehydrogenase (U/L) ≥250 vs. <250	1.891	1.189–3.007	0.007^**^	3.065	1.868–5.030	<0.001^***^
High‐risk cytogenetics^†^ Positive vs. negative	1.343	0.946–1.908	0.099	1.333	0.853–2.081	0.207
ASCT Yes vs. no	0.622	0.427–0.907	0.014^*^	0.415	0.231–0.744	0.003^**^
PH Yes vs. no	2.901	1.812–4.643	<0.001^***^	1.903	1.050–3.451	0.034^*^

Abbreviations: ASCT, autologous stem cell transplantation; CI, confidence interval; HR, harzard ratio; PH, pulmonary hypertension.

^†^ High‐risk cytogenetics: del(17p), t(4;14), and/or t(14;16).

^*^ *p* < 0.05

^**^ *p* < 0.01

^***^ *p* < 0.001.

## 4 DISCUSSION

Echocardiography is the most commonly used noninvasive assessment of PH and has been recommended in all clinical practice guidelines as the standard screening approach for patients with suspected PH.^18^ The prognostic significance of echocardiography‐defined PH in MM patients has been limitedly reported with controversial conclusions.^8^, ^9^ To our knowledge, this study is the largest cohort to specify the clinical characteristics and outcomes of MM patients with echocardiography‐defined PH. According to our results, baseline PH could independently predict shorter PFS and OS. Patients with PH were less likely to get deep and good responses. PH could be reversible with effective antimyeloma therapies, while it often recurred when MM relapsed.

Although PH is not a commonly recognized complication of MM, its relatively high prevalence rate during the course of MM has been reported in two retrospective studies.^8^, ^9^ However, the prevalence of baseline PH in NDMM has been rarely reported. In the present study, baseline PH at MM diagnosis could be identified in 12.7% of all 426 patients, showing a significantly higher incidence than in the entire adult population, which is reported as approximately 1%.^5^

In general, PH could be classified into five groups based on different pathophysiologic mechanisms according to World Health Organization (WHO) criteria.^19^ The specific mechanism of the relatively high prevalence rate of PH in MM remains to be clarified. Possible mechanisms could be summarized as renal insufficiency, left heart failure and other cardiac diseases (WHO group 2),^6^ pulmonary vascular amyloid deposition (WHO group 1),^20^, ^21^ pulmonary embolism (WHO group 4),^22^, ^23^ as well as treatment‐related adverse effect (WHO group 1).^10^, ^24^, ^25^ According to our results, PH is independent of the typical characteristics of MM such as M component type, stage and high‐risk cytogenetics, but is often accompanied by advanced age, anemia, and impaired renal function, which could contribute to inappropriately high cardiac output and cardiac insufficiency and ultimately promote left atrial hypertension and subsequent PH. Furthermore, higher baseline BNP/NT‐pro‐BNP levels and severe diastolic dysfunction were seen in PH patients, which have been reported to be related to poorer prognosis in MM.^26^, ^27^ These further confirmed the speculation that the WHO group 2 phenotype of cardiac dysfunction might play an important role in the mechanism of PH in MM.

The relationship between PH and thrombosis has also been explored. Although patients with PH presented with a higher D‐dimer level than those without PH, considering the fact that patients with PH had a significant older age, which has been proven to be related to increased D‐dimer levels,^28^, ^29^ the age‐adjusted D‐dimer cutoff value was used to for more accurate comparison.^17^ According to the result, after being adjusted by age, the positive incidence of D‐dimer did not show statistical differences. Moreover, the incidences of DVT and PE were comparable between the two groups, which further supported the hypothesis that WHO group 4 phenotype of pulmonary embolism was not the predominant mechanism in the development of PH in MM patients. Since patients with confirmed cardiac amyloidosis have been excluded from this study cohort, light chain amyloid deposition was not considered as a major mechanism of PH in our analysis, either. Nevertheless, the probability of localized amyloid deposition could not be totally excluded. According to the echocardiographic data, the LVPWD was higher in patients with PH. Although it has not reached the upper limit of the normal range, the possibility of early amyloidosis involvement and infiltrative cardiomyopathy should be considered.

With our results, AF was more frequently complicated in PH patients (9.3%). The high prevalence of AF in PH has been confirmed in multiple studies, with the incidence ranging from 3% to 31%.^30^, ^31^, ^32^ According to the literature, AF is mainly considered to be secondary to PH. Although the specific mechanism is still unclarified, one main pathophysiologic explanation is that increased PAPs would cause increased pressure and volume loads on the right heart, inducing right atrial remodeling and then leading to AF.^33^ Nevertheless, left heart dysfunction (WHO group 2) and thrombosis embolism (WHO group 4) caused by AF could also be the etiology of PH. Based on our data, we tend to interpret the high prevalence of AF as the combined effect of PH and cardiac dysfunction.

Our results also confirmed that PH could be reversible with effective treatment of MM, which is usually accompanied by improved diastolic dysfunction and normalized BNP/NT‐pro‐BNP levels, while might reoccur when MM relapse. These findings further support our speculation that PH is secondary to MM rather than an independent pathological process in MM patients. According to several published case reports, besides MM, reversible PH could be also seen in other plasma disorders including smoldering MM, plasma cell leukemia, and POEMS syndrome (characterized by polyneuropathy, organomegaly, endocrinopathy, monoclonal protein, and skin changes).^34^, ^35^, ^36^, ^37^ The common feature of these cases indicates that PH is usually associated with active disease status. The dysregulation of specific cytokines, such as vascular endothelial growth factor, interleukin‐6, interleukin‐1β, tumor necrosis factor‐α, or transforming growth factor‐α, has been presumed as a possible mechanism of PH development in these settings.^37^, ^38^, ^39^, ^40^, ^41^ However, these findings are limited to case reports and thus could not be considered convincing evidence. Further assessment of the biomarkers and cytokines in large population‐based studies might provide further understanding of the pathophysiology of PH development in specific populations.

As to the aspect of MM treatment, our results revealed that PH could affect the depth of response of MM patients. Even though with similar intensity of treatment, patients with PH are still less likely to get deeper responses than those without PH at MM diagnosis. Our results also confirmed the adverse prognostic effect of PH on both PFS and OS in NDMM patients, which is consistent with a previously published retrospective study.^8^ This adverse effect is independent of general prognostic factors of MM such as ISS stage, high‐risk cytogenetics, LDH levels, and ASCT, which demonstrates the important additional prognosis value of baseline echocardiography‐defined PH in MM patients. Therefore, optimal treatment for these patients should be further evaluated. More effective and less cardiotoxic therapies need to be explored. While some first‐line antimyeloma agents, including carfilzomib, bortezomib, and thalidomide, have also been reported to contribute to PH with a very low probability, close monitoring of echocardiography would be recommended for PH patients during treatment. As PH is usually reversible with effective induction therapies with improved cardiac function, ASCT is still a strong recommendation for eligible patients.

There are also some limitations in this study that should be considered. One main limitation is that echocardiography data were retrospectively collected and were nonspecific for PH. Parameters of right cardiac function were not completed, resulting in an unevaluable diastolic dysfunction grade in some patients. Another limitation is that a complete series of echocardiography monitoring at different disease statuses was not available in part of the patients. Larger prospective studies are needed to further clarify the association between the degree of PH improvement and clinical prognosis.

In conclusion, the current study demonstrates the important prognostic value of echocardiography‐defined PH in MM patients and should be routinely evaluated in MM patients at diagnosis to make a precise prognosis.

## CONFLICT OF INTEREST

All authors declare that they have no conflict of interest.

## AUTHORS’ CONTRIBUTIONS

WG and WC designed this study. YJ, HZ, GY, CG, and YT provided study patients and collected data. YJ, YW, and ZZ performed data analysis. YJ, HZ, and YW wrote the manuscript. WG and WC supervised the study.

## ETHICS STATEMENT

This study was approved by the Ethics Committee of Beijing Chaoyang Hospital.

## Data Availability

Research data are not shared.
